# Number of Births and Later-Life Depression in Older Adults: Evidence from China

**DOI:** 10.3390/ijerph191811780

**Published:** 2022-09-18

**Authors:** Kaiyun Xue, Yafeng Nie, Yue Wang, Zhen Hu

**Affiliations:** 1 College of Economics and Management, Northwest A&F University, Yangling, Xianyang 712100, China; 2 School of Economics, Jinan University, Guangzhou 510632, China

**Keywords:** fertility behavior, depressive symptoms, abortions, gender differences, China

## Abstract

Previous studies on the number of births and the health of the elderly have been highly focused on physical health and used samples from developed countries. Therefore, this study aimed to explore the effect of the lifetime number of births on depression in Chinese older adults. We used panel data for men and women aged 50 and over from the 2013–2018 China Health and Retirement Longitudinal Study. Depressive symptoms were assessed through a short form of the Center for Epidemiologic Studies Depression Scale. We found that depression levels in women were significantly higher than in men, with a mean difference of 2.44 (*p* < 0.001). The model estimation results indicated that the number of births affected depression in older adults, and its increase could exacerbate depression in older adults. The number of births significantly impacted depression among the elderly aged 50–69. Furthermore, there was a negative relationship between the proportion of sons and older adults’ depression, which was significant in older males; the number of abortions may exacerbate depression in older adults, especially in females.

## 1. Introduction

The impact of events across the life course on the health of older adults has received widespread academic attention. Studies have found that reproductive history (e.g., age of first childbirth and number of births) may have implications for health in later life [1,2,3]. In China, to actively respond to population aging, fertility policies have undergone changes, from the former one-child policy (1979–2015) to the subsequent universal two-child policy (2015–2021), which may bring about a shift in fertility patterns and directly affect the number of births in families. It raises the question of whether the number of births affects the health of the elderly. Theoretically, from an evolutionary biology perspective, fertility behaviors significantly impact on the health of organisms. “Disposable soma theory” and the theory of “maternal depletion” suggest that women with more children generally have poorer health, such as metabolic syndrome, obesity, diabetes, cardiovascular disease, and certain cancers [4,5,6]. Among men, those with a high level of fertility are also in poorer physical health [6]. An empirical study from Sweden showed that the relationship between having children and mortality follows a U-shaped pattern where childless parents and parents with one or 4+ children have elevated mortality [7]. Another study reported a J-shaped relationship between having children and the risk of cardiovascular disease [8]. In contrast, having more children may also have health benefits for women, such as preventing uterine, ovarian, and breast cancers [1]. In addition, the relationship between fertility behaviors and mental health has been investigated in several studies, with mixed results [9,10,11,12]. A study by Li et al. found that more full-term pregnancies and more incomplete pregnancies are related to a higher prevalence of depressive symptoms [13]. Based on Korean data, a study by Kim et al. discovered that having offspring has a large effect on the prevalence of depressive disorder, and fathers are at lower risk for depressive disorder than mothers [14]. Grundy et al. used nationally representative longitudinal data from England to confirm the important influence of fertility history on depression in older age groups [15]. In China, studies found an association between fertility behavior and depression among the elderly [12,16]. There is a sharp rural–urban divide in the relationship between parental status and depression [17]. Given the different roles that fathers and mothers play in childbirth and parenting, fertility behaviors can be more likely to impact mothers’ mental health [18,19]. There are significant gender differences in the psychological impact of lifetime childlessness. A study by Dykstra and Wagner found no differences in life satisfaction between women who had never had children and women whose children had all survived [20], while men’s life satisfaction was negatively associated with lifetime childlessness [21]. Because of gender-biased filial expectations and strong son preference in China, the gender structure of children is associated with the mental health of older adults [17]. Meanwhile, several studies proposed that abortions can have effects on mental health, such as a possible increased risk of subsequent depression in women undergoing an induced abortion [22], as well as the long-lasting adverse psychological effects of spontaneous abortion [23].

With the increasing population aging in China, the mental health of the elderly is becoming increasingly prominent. A survey by the National Health Commission of the People’s Republic of China showed that less than one in three older adults have good mental health. This phenomenon is affected by many factors, including rapid urbanization, the level of mental health services, and changes in fertility policies [24,25]. In terms of fertility policy, the outcome of the two-child policy will eventually be more elderly parents benefiting from the care of daughters, thus enhancing their mental and physical health [25]. China’s new fertility policy, introduced in 2015, permits all couples to have two children, up from one previously, which may cause a change in the number of births in families. In the context of the low development of social retirement programs and financial markets, adult children can also provide financial support and daily care for the elderly, particularly in rural China. As a result, people think that the more children they have, the more secure their later life will be, which is consistent with the traditional Chinese concept of more children being equal to more blessings [26,27].

Previous research has focused on the effects of fertility behaviors on the physical health of older adults, such as longevity and chronic disease, and less on mental health. Furthermore, they used samples from developed countries, such as those in Europe, while fewer studies focused on Chinese data. Given China’s special fertility policy and traditional attitudes, the impact of the number of births on the mental health of older adults still needs further investigation. This study analyzed nationally representative longitudinal data from China to examine the association between the number of births and later-life depression and explore whether or not the association differs by gender and age. Furthermore, we also analyzed the effect of the proportion of sons and abortions on depression in the elderly.

## 2. Materials and Methods

### 2.1. Participants

This study used data from the national follow-up survey of CHARLS conducted by the National School of Development, Peking University. The national baseline survey was conducted in 2011; participants aged over 45 years old were interviewed every two years [28]. As of 2018, CHARLS had conducted four national follow-up surveys of over 12,400 sample households and 19,000 respondents in 150 counties and 450 communities or villages in 28 provincial administrative regions. Moreover, CHARLS also conducted a life history survey in 2014, which included information such as pregnancy history. Our study used the matched data of the latest three follow-up surveys and the life history survey and selected respondents aged 50 years and above. After excluding missing values, the final sample size was 10,156 observations with 5593 respondents. The data matching and selection process resulted in many missing values. Moreover, CHARLS did not allow elderly respondents to have family members answer about their mental health status if they could not, which resulted in a large number of missing data for the key outcome variable of depression.

### 2.2. Measures

#### 2.2.1. Outcome Variable

Following Andresen et al., a short form of the Center for Epidemiologic Studies Depression Scale (CES-D) was used to measure the depression level of the respondents in this study [29]. The CHARLS questionnaire used a 10-item depression scale to measure the depression of the elderly in the past week [30]. It consisted of three items relating to depression, five items relating to somatic symptoms, and two items relating to positive emotions, with four response options: (1) rarely or none of the time (<1 day); (2) some or a few times (1–2 days); (3) occasionally or a moderate number of times (3–4 days); (4) most or all of the time (5–7 days). The option values for the depression and somatic symptom items were sequential integers from 0 to 3, while the options for the positive emotions items were assigned values in reverse. Their scores were summed to obtain the individual depression level. The higher the score, the more depressed the respondent. In addition, an ordered categorical variable was created to define the degree of depression (for sample characteristic statistics). Based on the criteria for depression among the elderly established through the CES-D-10 scale, different individual values were assigned as follows: (1) a value of 1 was assigned to individuals with scores ranging from 0 to 5, (2) a value of 2 was assigned to individuals with scores ranging from 6 to 11, (3) a value of 3 was assigned to individuals with scores ranging from 12 to 17, (4) a value of 4 was assigned to individuals with scores ranging from 18 to 23, (5) a value 5 of was assigned to individuals with scores ranging from 24 to 30. 

#### 2.2.2. Explanatory Variable

The number of births was the core explanatory variable in this study, which is the number of children parents have had. Respondents were asked, “How many livings biological children do you have?”, and we added the number of deceased biological children to obtain the number of births in this study. This study also examined the effect of other fertility behavior and abortion on depression, including the proportion of sons and the number of abortions. The former variable is the ratio of sons to total births (excluding the number of abortions), and the latter is the number of induced and natural abortions.

#### 2.2.3. Control Variable

This study controlled for individual and family characteristics, including age, gender, spouse status, residential area, educational status, financial assets, work status, pension, cohabitation with children, smoking status, drinking status, and abortion history. Further, we also controlled for psychological treatment with one lag period (psychological treatment (lagged one period) indicates whether the respondent received treatment for their emotional, nervous, or psychiatric problems in the previous wave of the questionnaire) in addition to individual and family characteristics. Among these, age was a continuous variable, and the lower limit was set to 50 years. Gender, spouse status, residential area, work status, pension, cohabitation with children, smoking status, drinking status, and psychological treatment (lagged one period) were set as dummy variables which were assigned the following values: gender (1: female; 0: male), spouse status (1: available; 0: not available), residential area (1: urban; 0: rural), work status (1: available; 0: not available), pension (1: covered; 0: not covered), cohabitation with children (1: yes; 0: no), smoking status (1: yes; 0: no), drinking status (1: yes; 0: no), abortion history(1: yes; 0: no), and psychological treatment with one lag period (1: yes; 0: no). Educational status was set as a categorical variable (1: less than lower secondary; 2: upper secondary and vocational training; 3: tertiary). Financial assets were measured by summing personal savings, stocks, funds, and bonds and taking the logarithmic value.

### 2.3. Data Processing and Analysis

Stata 15.0 was used to analyze the data. Sample characteristic statistics were calculated for most variables used in this study. Independent sample *t*-tests were used to assess the differences in mean for each variable between males and females. The panel data set for this study covered three years: 2012, 2015, and 2018. It allowed the elimination of the influence of unobservable variables to reduce bias. Fixed-effects models, random-effects models, and pooled ordinary least squares (OLS) are common statistical methods for analyzing panel data. The fixed-effects models can only estimate the effect of the independent variable over time on the dependent variable, while the random-effects models can estimate the effect of time-invariant individual characteristics on the dependent variable. Therefore, some studies believe that the random-effect model is more applicable than the fixed-effect model in some cases [31]. To test the effects of the number of births, proportion of sons, and the number of abortions on depression in older adults, statistical analyses were carried out using random-effect generalized least square (GLS) regression. The model was set up as follows:

(1) $$Depression_{it}=\beta_{0}+\beta_{1}Fertility_{it}+\beta_{2}x_{2it}+\ldots +\beta_{k}x_{kit}+\zeta_{i}+\varepsilon_{it}$$

where i is the subscript of the individual, and t is the subscript of the time. $Depression_{it}\;$ is the observed value of the dependent variable for individual i at time point t. $Fertility_{it}$ is the explanatory variable, including the number of births, the proportion of sons, and the number of abortions. $x_{2it}$ to $x_{kit}$ represent control variables, including individual and family characteristics, etc. $\xi_{i}$ is the random heterogeneity specific to the ith individual and is time invariant. $\varepsilon_{it}$ is the error term.

## 3. Results

### 3.1. Basic Characteristics of the Participants

A total of 10,156 participants (3783 males and 6373 females) were included in this study. Table 1 summarizes the general characteristics of the final sample. In terms of the outcome variable, depression levels were significantly higher in women, with a mean depression value of 9.30 in female participants compared to 6.86 in male participants, with a mean difference of 2.44, which was significant at the 1% statistical level. In terms of fertility behavior characteristics, participants with three or more births accounted for more than 50% of the sample. The number of abortions was 0 among most participants (75.43%), without obvious gender differences. The majority of participants had sons, accounting for 87.89% of the total sample. In terms of personal characteristics, 76.01% of the participants were married, and the percentage of participants with rural residences was 63.51%. The overall education level of the participants was low, with 91.21% having lower than junior high school education. More than 40% of the male participants smoked and drank alcohol.

### 3.2. The Relationship between Number of Births and Depression

**Figure 1:**
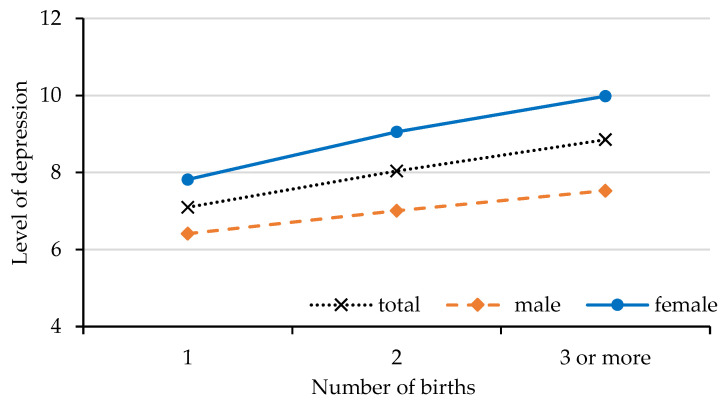
Number of births and depression in the elderly.

First, we statistically analyzed the correlation between the number of births and depression in older adults (Figure 1: Number of births and depression in the elderly). Our results showed that the level of depression in the elderly increased gradually with the number of births. This trend was generally consistent across the full sample, male respondents and female respondents, but the depression level was significantly higher in women than in men, and the mean depression level increased more in older women than in men as the number of births increased. Second, we examined the impact of the number of births on depression in the elderly; the results are shown in Table 2. Model 1 reports the results for the full sample, and the results show that the coefficient of the overall effect of the number of births on depression in the elderly was 0.245. The coefficient of the effect of the number of births on depression in the elderly was 0.245, which was significant at the 5% statistical level, indicating that the number of births increases the likelihood of depression in the elderly. The results of Model 2 and Model 3 show that the number of births has a negative effect on depression in men, but it is an important factor that causes depression in older women.

As shown in Table 2, in addition to the core explanatory variable of the number of births, among the control variables, depression decreased significantly with age (Model 1), and older adults had a more optimistic and positive attitude and tended to have better mental health, as described by the Chinese proverb, “At 30, I found my balance through the rites. At 40, I was free from doubts about myself. At 50, I understood what Heaven intended me to do. At 60, I was attuned to what I heard. At 70, I followed what my heart desired without overstepping the line”. In terms of spouse status, married older adults were less depressed. Compared with men, female older adults were more depressed, which may be related to the fact that women have been more engaged in family care and lacked social support for a long time, so the psychological condition of older women is relatively bad. Compared to urban older adults, rural older adults showed higher levels of depression, which may be related to the fact that the development of social security and medical coverage in rural areas lags behind that in urban areas. There was a negative relationship between educational status, financial assets, work, and depression among older adults.

**Figure 2:**
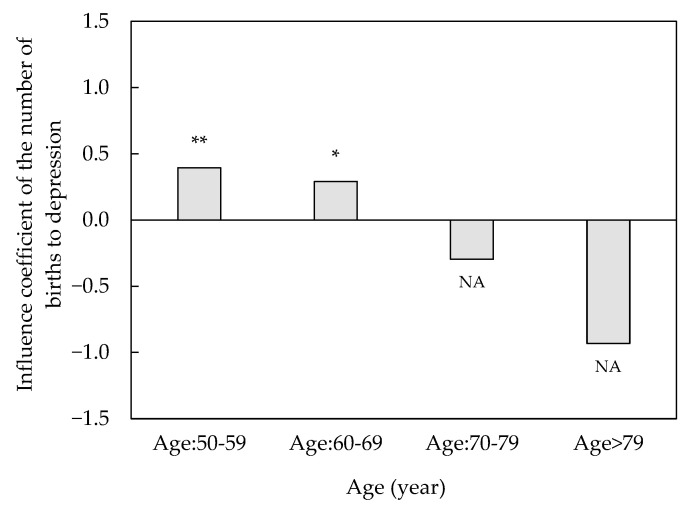
Number of births and depression in different age groups. Notes: * *p* < 0.05, ** *p* < 0.01; NA stands for *p* > 0.1.

The mental health of older adults is the result of cumulative social, physical, and psychological effects experienced over time and may vary according to individual characteristics. To analyze the effect of the number of births on depression in different age groups in more detail, we divided the samples into four groups by the age of respondents, and the results are shown in Figure 2. From the results of the age subsample, the number of births only significantly increased the level of depression in the elderly aged 50–69, while it did not have a significant effect on the elderly group aged 70 and above. More specifically, the number of births had the largest and most significant effect on the severity of depression among older adults aged 50–59 years.

### 3.3. The Relationship between the Proportion of Sons and Depression

In addition to the number of births, fertility behaviors such as birth gender structure have an impact on the health of older adults. This study further examined the effects of the proportion of sons on depression in older adults, and the results are shown in Table 3: Proportion of sons and depression. In the full sample, the proportion of sons was associated with lower levels of depression in older adults, but the negative effect was not significant. The results of Model 5 and Model 6 showed that there was a significant negative correlation between the proportion of sons and depression in the male elderly. At the same time, it had no significant negative effect on depression in the female elderly.

### 3.4. The Relationship between Abortion and Depression

Table 4 shows the association between abortion and depression in the full sample (Model 7), male (Model 8), and female groups (Model 9). The results demonstrated that the higher the number of abortions, the more likely the elderly were to be depressed. The coefficient of the effect of the number of abortions on the level of depression in older women was 0.442 and was significant at the 5% statistical level, whereas the effect on depression in older men was insignificant, and the coefficient was smaller than in the female sample.

## 4. Discussion

Using data from the CHARLS database, this study examined the relationship between the number of births and depression among older adults in China and analyzed the differences in the effects of births by gender and age. Furthermore, we also explored how the proportion of sons and the number of abortions affect depression in the elderly.

This study found that the number of births can significantly affect the level of depression in older adults. The biological response to childbearing is associated with the risk of chronic disease in later life, and women with more births tend to have poorer health. Furthermore, the financial stress of raising children, the crowding out of personal leisure time, and the impact of too much family care on career development increase with the number of births, which causes both physical health problems and increased mental stress, with a significantly increased probability of depression in older adults. As the number of births increases, the number of physical health problems for the individual increases, which increases the level of depression in older adults.

In addition, our results showed that the number of births has a greater effect on women’s mental health status than that of older men, which is consistent with the findings of existing studies [32]. As a unique event experienced by women, women with more births have lower lifetime levels of circulating estrogen compared to women with fewer or no births [33], and estrogen usually has antidepressant effects, so having more births directly increases women’s depression levels. Meanwhile, our result demonstrated that the number of births had the largest and most significant effect on depression among older adults aged 50–59 years. This age group experienced the period of family planning policy in China (In March 1978, the constitution stipulated that “the state advocates and promotes family planning”. In September 1982, the 20th National Congress of the Communist Party of China established family planning as a basic state policy) which advocated that a couple should have only one child, and, if a second child was born without approval, it was considered a violation of family planning regulations, and the family was punished accordingly, such as through the loss of jobs. Families with strong gender preference risked unemployment, fines, and other risks and costs to continue giving birth if their first child was a girl. The greater the number of births, the higher the cost of childbirth the family bears, and the more likely it is to cause depression. Meanwhile, under the idea of “having a son to carry on his family name”, if there is only one daughter in the family, parents face greater social pressure. In the context of such a policy, the early experience of being punished with fines and other penalties for having a large number of births, or the long-term social pressure, may have increased the likelihood of depression in old age. Further, there was a marginal decreasing effect of the number of births on older adults’ depression among those over 70. Since the children of the elderly over 70 have independent financial ability, they can provide financial support for their parents. The increase in births gives parents greater access to their children [34], and children have more contact with their parents and provide them with financial support. This strong contact increases parents’ well-being in later life and, to some extent, attenuates the degree of depression in older adults.

Our study further examined the effects of the proportion of sons and abortion on depression in older adults. The proportion of sons was negatively correlated with depression in the elderly, and it was significant in the male group. This may be related to the traditional Chinese beliefs of “son preference” and “raising sons for old age”, as well as the fact that older adults rely more on daughters for caregiving, and those with daughters tend to be more satisfied with their life and health status; the proportion of sons in the full sample had no significant effect on depression in older adults. Further, the idea of “having a son to carry on his family name” is more common in Chinese men, which may be why the proportion of sons significantly reduced depression in older men. Whether induced or natural, an abortion causes damage to a woman’s body, so early abortion experience affects more women. This is because both induced and spontaneous abortions can cause harm to women’s health.

This study had some limitations that should be noted. First, due to data limitations, this study did not directly incorporate fertility policy as a variable into the research model to examine the effect of the number of births on depression in the elderly under various fertility policies. Furthermore, we assessed the level of depression by the CES-D-10. Although the CES-D-10 is widely used in the study of depression in older adults, it is not a clinical diagnosis. Finally, this paper only examined the effect of fertility behaviors on depression in older adults, and we suggest that the effect of grandchildren could be considered in future studies of depression in older adults.

## 5. Conclusions

Based on data from the latest three follow-up surveys and the life history survey from CHARLS, this study examined the effect of the number of births on depression among older adults using a random-effects model. First, our results suggest that the number of births significantly impacts depression in older adults and that its increase can exacerbate depression in older adults. In addition, the effect is greater among women. Second, our results show that the effect of the number of births on depression was significantly greater for older adults aged 50–69 but had no significant effect for those aged 70+. Finally, we analyzed the effects of the proportion of sons and the number of abortions on older adults’ depression. There was a negative relationship between the proportion of sons and older adults’ depression, and it was significant in older males; the number of abortions may exacerbate depression in older adults, especially in females. Therefore, on the one hand, when liberalizing the fertility policy to encourage people to have more children, it is necessary to continuously improve the supporting policies related to fertility, such as improving the level of reproductive health and maternal and child health care and improving the maternity leave and insurance system for women to alleviate the adverse physiological and psychological effects of fertility on women. On the other hand, we should actively respond to the aging of the population, establish a sound social security system for the elderly, and build a system of elderly care services that is coordinated with home and community institutions and combined with medical and health care.

## Figures and Tables

**Table 1:** Sample characteristic statistics.

Characteristics	Total		Male		Female		Differences in Mean
	n	%	n	%	n	%	
**Degree of depression**							−2.44 ***
0–5	3898	38.38	1808	47.79	2090	32.79	
6–11	3436	33.83	1273	33.65	2163	33.94	
12–17	1746	17.19	469	12.40	1277	20.04	
18–23	802	7.90	197	5.21	605	9.49	
24–30	274	2.70	36	0.95	238	3.73	
**Number of births**							−0.10 ***
1	1126	11.09	478	12.64	648	10.17	
2	3203	31.54	1317	34.81	1886	29.59	
3 or more	5827	57.37	1988	52.55	3839	60.24	
**Number of abortions**							0.02
0	7661	75.43	2804	74.12	4857	76.21	
1	2175	21.42	872	23.05	1303	20.45	
2	301	2.96	97	2.56	204	3.20	
3 or more	19	0.19	10	0.26	9	0.14	
**Number of sons**							−0.10 ***
0	1230	12.11	511	13.51	719	11.28	
1	4218	41.53	1607	42.48	2611	40.97	
2	3057	30.10	1157	30.58	1900	29.81	
3 or more	1651	16.26	508	13.43	1143	17.94	
**Age (year)**							−0.05 *
50–59	2705	26.63	1001	26.46	1704	26.74	
60–69	4523	44.54	1756	46.42	2767	43.42	
70–79	2353	23.17	864	22.84	1489	23.36	
>79	575	5.66	162	4.29	413	6.48	
**Spouse status**							0.29 ***
Not available	2436	23.99	209	5.52	2227	34.94	
Available	7720	76.01	3574	94.48	4146	65.06	
**Residential area**							0.01
Rural	6450	63.51	2378	62.86	4072	63.89	
Urban	3706	36.49	1405	37.14	2301	36.11	
**Educational status**							0.11 ***
Less than lower secondary	9263	91.21	3227	85.30	6036	94.71	
Upper secondary and vocational training	789	7.77	482	12.74	307	4.82	
Tertiary	104	1.02	74	1.96	30	0.47	
**Financial assets**							0.10 ***
Below average	7303	71.91	2481	65.58	4822	75.66	
Average or above average	2853	28.09	1302	34.42	1551	24.34	
**Work status**							0.15 ***
No	4319	42.53	1250	33.04	3069	48.16	
Yes	5837	57.47	2533	66.96	3304	51.84	
**Pension**							−0.02 ***
No	3586	35.31	1386	36.64	2200	34.52	
Yes	6570	64.69	2397	63.36	4173	65.48	
**Cohabitation with children**							−0.03 ***
No	5675	55.88	2196	58.05	3479	54.59	
Yes	4481	44.12	1587	41.95	2894	45.41	
**Drinking status**							0.40 ***
No	7283	71.71	1757	46.44	5526	86.71	
Yes	2873	28.29	2026	53.56	847	13.29	
**Smoking status**							
No	7945	78.23	1914	50.59	6031	94.63	0.44 ***
Yes	2211	21.77	1869	49.41	342	5.37	
**Psychological treatment (lagged one period)**							
No	10,139	99.83	3778	99.87	6361	99.81	−0.00
Yes	17	0.17	5	0.13	12	0.19	
**Year**							
2013	2328	22.92	599	15.83	1729	27.13	0.43 ***
2015	3811	37.52	1525	40.31	2286	35.87	
2018	4017	39.55	1659	43.85	2358	37	

Notes: * *p* < 0.05, *** *p* < 0.001.

**Table 2:** Number of births and depression.

Variables	Model 1	Model 2	Model 3
	Total	Male	Female
	β (S.E)	β (S.E)	β (S.E)
Number of births	0.245 ** (0.117)	−0.059 (0.163)	0.386 ** (0.160)
Age	−0.020 * (0.011)	0.032 * (0.017)	−0.031 ** (0.014)
Spouse status	−1.191 *** (0.176)	−0.787 ** (0.375)	−0.799 *** (0.221)
Residential area	−1.421 *** (0.162)	−1.276 *** (0.229)	−1.529 *** (0.219)
Educational status	−1.294 *** (0.227)	−1.069 *** (0.255)	−0.937 ** (0.397)
Financial assets	−0.205 *** (0.021)	−0.140 *** (0.032)	−0.227 *** (0.027)
Work status	−0.331 ** (0.137)	−0.429 ** (0.212)	−0.160 (0.176)
Pension	0.222 (0.137)	0.259 (0.210)	0.119 (0.177)
Cohabitation with children	−0.164 (0.123)	0.334 * (0.180)	−0.420 ** (0.163)
Drinking status	−0.788 *** (0.149)	−0.624 *** (0.189)	−0.008 (0.243)
Smoking status	−0.430 ** (0.171)	0.365 * (0.196)	0.777 * (0.408)
Psychological treatment (lagged one period)	2.476 * (1.382)	0.752 (2.409)	3.040 * (1.690)
Abortion history	0.425 ** (0.172)	0.234 (0.241)	0.473 ** (0.235)
Constant	13.855 *** (0.798)	8.676 *** (1.242)	14.133 *** (1.103)
Observations	10,156	3783	6373

Notes: * *p* < 0.05, ** *p* < 0.01, *** *p* < 0.001. Standard error of estimated coefficients is shown in parentheses.

**Table 3:** Proportion of sons and depression.

Variables	Model 4	Model 5	Model 6
	Total	Male	Female
	β (S.E)	β (S.E)	β (S.E)
Proportion of sons	−0.302 (0.234)	−0.643 ** (0.323)	−0.013 (0.323)
Age	−0.013 (0.010)	0.030 * (0.016)	−0.019 (0.014)
Spouse status	−1.192 *** (0.176)	−0.793 ** (0.375)	−0.787 *** (0.221)
Residential area	−1.476 *** (0.160)	−1.261 *** (0.226)	−1.610 *** (0.217)
Educational status	−1.344 *** (0.226)	−1.074 *** (0.255)	−1.056 *** (0.395)
Financial assets	−0.209 *** (0.021)	−0.137 *** (0.032)	−0.232 *** (0.027)
Work status	−0.317 ** (0.137)	−0.421 ** (0.212)	−0.150 (0.176)
Pension	0.216 (0.137)	0.267 (0.210)	0.108 (0.177)
Cohabitation with children	−0.146 (0.123)	0.336 * (0.180)	−0.401 ** (0.163)
Drinking status	−0.794 *** (0.149)	−0.612 *** (0.188)	09 (0.243)
Smoking status	−0.439 ** (0.171)	0.369 * (0.196)	0.782 * (0.408)
Psychological treatment (lagged one period)	2.472 * (1.382)	0.701 (2.407)	2.959 * (1.690)
Abortion history	0.392 ** (0.172)	0.222 (0.240)	0.436 * (0.235)
Constant	14.240 *** (0.803)	8.993 *** (1.249)	14.524 *** (1.106)
Observations	10,156	3783	6373

Notes: * *p* < 0.05, ** *p* < 0.01, *** *p* < 0.001. Standard error of estimated coefficients is shown in parentheses.

**Table 4:** Number of abortions and depression.

Variables	Model 7	Model 8	Model 9
	Total	Male	Female
	β (S.E)	β (S.E)	β (S.E)
Number of abortions	0.329 ** (0.142)	0.078 (0.202)	0.422 ** (0.192)
Age	−0.013 (0.010)	0.030 * (0.016)	−0.019 (0.014)
Spouse status	−1.188 *** (0.176)	−0.789 ** (0.375)	−0.785 *** (0.221)
Residential area	−1.475 *** (0.160)	−1.246 *** (0.227)	−1.615 *** (0.217)
Educational status	−1.339 *** (0.226)	−1.050 *** (0.254)	−1.060 *** (0.394)
Financial assets	−0.209 *** (0.021)	−0.137 *** (0.032)	−0.232 *** (0.027)
Work status	−0.321 ** (0.137)	−0.437 ** (0.212)	−0.148 (0.176)
Pension	0.215 (0.137)	0.260 (0.210)	0.105 (0.177)
Cohabitation with children	−0.151 (0.123)	0.324* (0.180)	−0.400 ** (0.163)
Drinking status	−0.789 *** (0.149)	−0.617 *** (0.188)	0.011 (0.243)
Smoking status	−0.439 ** (0.171)	0.369 * (0.196)	0.767 * (0.408)
Psychological treatment (lagged one period)	2.469 * (1.382)	0.794 (2.408)	2.941 * (1.690)
Constant	14.085 *** (0.792)	8.656 *** (1.236)	14.511 *** (1.092)
Observations	10,156	3783	6373

Notes: * *p* < 0.05, ** *p* < 0.01, *** *p* < 0.001. Standard error of estimated coefficients is shown in parentheses.

## Data Availability

Data were obtained from the CHARLS and are available [http://charls.pku.edu.cn] (accessed on 11 December 2021) with the permission of the CHARLS.
